# A retrospective evaluation of bone graft success, implant survival rate and marginal bone loss

**DOI:** 10.6026/973206300211534

**Published:** 2025-06-30

**Authors:** Laxman Singh Kaira, Virag Srivastava, Sauvik Mazumdar, Ishita Mudgal, Karandeep Singh, Ashish Prajapati, Megha Patel

**Affiliations:** 1 Department of Dentistry, GMC, Datia, Madhya Pradesh, India; 2 Department of Dentistry, Government Medical College, Haldwani, Uttarakhand, India; 3 Department of Dentistry, JMN Medical College and Hospital, Chakdaha, Nadia, West Bengal, India; 4 Department of Prosthodontics, Shri Aurobindo College of Dentistry, Indore, Madhya Pradesh, India; 5 Department of Oral & Maxillofacial Surgery, SGT University, Budhera Village, Gurugram, Haryana, India; 6 Department of Oral & maxillofacial surgery, Kalka dental college, Meerut, Uttar Pradesh, India; 7 Department of Pediatric and Preventive Dentistry, Karnavati School of Dentistry, Karnavati University, Gandhinagar, Gujarat, India

**Keywords:** Bone graft, dental implants, implant survival, marginal bone loss, osseointegration, graft success

## Abstract

Bone grafting serves to restore the alveolar bone defect, providing adequate alveolar bone essential for long-term implant survival. Therefore, it is of interest to evaluate bone graft success, implant survival rate and marginal bone loss. Hence, data from 85 patients showed 112 implants performed in grafted sites achieved a 95.5% survival rate and the bone grafting success reached 92.8%. Auto-genous grafts displayed higher functionality than allografts and xenografts did. At 12 months the mean marginal bone loss measured 1.12± 0.28 mm according to graft classification and implant placement site (p<0.05). Bone grafting shows its effectiveness for improving implant stability and achieving enduring success within patients who need large bone grafts.

## Background:

Bone grafting serves to restore the alveolar bone defect, providing adequate alveolar bone essential for long-term implant survival [1]. The complete placement and stability of dental implants remains difficult when patients lack enough alveolar bone volume. Ineffective bone maintenance post-tooth loss or trauma forces medical practitioners to conduct bone augmentation surgeries for achieving proper implant placement [2, 3]. Ridge reconstruction for bone deficiency requires various treatment components which use autogenic bone grafts in combination with allografts or xenografts or alloplastic materials. Autogenous bone stands as the preferred choice for bone grafts because it possesses osteogenic and osteoinductive and osteoconductive properties yet its availability is limited by donor site morbidity issues [4, 5]. Patients determine the success rate of graft procedures through tests of tissue integration paired with clinical stability effectiveness and long-term implant support maintenance [6]. Treatment success evaluation requires assessment of implant survival together with marginal bone loss measurements. Bone loss around implants known as MBL manifests during the first year of implant placement due to natural tissue reactions and various factors that affect the results [7, 8]. Therefore, it is of interest to evaluate bone graft success, implant survival rate and marginal bone loss.

## Implant therapy needs two fundamental components:

For success it requires both osseointegration and protected integrity of the bone and soft tissue at the implant site. The passage of time reveals marginal bone reduction particularly around implants installed in grafted locations which leads to poor esthetics and advances peri-implant disease and final implant failure [3, 4]. Studies that estimate both implant survival rates and marginal bone loss offer complete insight into long-term success of implant-supported rehabilitations. The evaluation of graft materials regarding their bone loss patterns enables clinicians to make patient-specific decisions based on scientific evidence [5]. The selection of bone graft materials for clinical decisions depends on multiple factors including the implant site number, location of the defect and patient choice and repair costs and experience level of the practitioner. The use of autogenous bone as a material provides excellent outcomes nevertheless donors faces complications and procedure-related limitations of supply. Xenografts and allografts gained popularity as replacement solutions because they offer both patient-friendly characteristics along with minimal adverse effects [6]. Modern technology has produced synthetic along with bioactive materials which aim to replicate natural bone while showing different results in clinical applications. Retrospective studies designed with proper comparison methods enable researchers to determine the benefits and limitations of the available implant options [7]. The stability of dental implants depends greatly on where they are placed in the mouth between anterior and posterior positions and where they are located between maxilla regions and mandible along with immediate versus delayed loading times. Literature research indicates that implant success rates depend on anatomy-related factors and bone density levels and the way teeth bite against each other [8]. The outcome assessment of implemented grafts along with implant survival rates requires analysis of these elements in connection with the selected graft materials. This research employs retrospective data evaluation to advance knowledge about the united effects between factors that affect implant therapy outcomes in grafted areas [9]. This research checks the bone graft success rate before implant placement and analyzes the dental implant survival rate along with measuring their marginal bone deterioration throughout the specified follow-up period through retrospective analysis.

## Materials and Methods:

## Study design and sample selection:

Medical records together with X-ray images provided data about patients who received bone grafts then received dental implants from January 2018 to December 2021. Research included adult patients between 25 to 65 years who underwent bone grafting procedures using autograft or allograft or xenograft followed by endosseous dental implant placement having at least 12 months of post-loading treatment. Patients with systemic bone healing disorders including uncontrolled diabetes and bisphosphonate therapy or medical history of radiotherapy in the head and neck area or poorly documented cases were excluded from the study.

## Surgical procedure:

Local anesthesia with standard aseptic protocols served as the basis for conducting all grafting surgical procedures. The site-specific defect sizes required grafting materials that included autogenous bone from mandibular ramus or symphysis along with DFDBA or bovine-derived xenograft. The healing period lasted 4-6 months and then dental implants received standard placement following surgical procedures. Healing abutments served as implants were applied after the osseointegration period before the prosthetic treatment commenced.

## Data collection:

A review of patient records contained data about demographics and implant dimensions together with implant placement sites and healing periods and follow-up results. The definition for implant survival required no mobility and no pain and no infection along with the implant operating successfully. The study evaluated bone graft success by the integration of graft tissue and by confirming both no exposure and no graft loss.

## Radiographic evaluation:

Standardized periapical radiographs obtained through paralleling technique helped evaluate marginal bone levels at three time points: implant placement and prosthesis placement and the one-year post-loading period. The calibrated digital software measured implant platform and first bone-to-implant contact distance on both mesial and distal aspects. The laboratory measured marginal bone loss through calculating an average of both recorded readings.

## Statistical analysis:

The analysis was conducted through Microsoft Excel and SPSS software (version 26, IBM Corp., Armonk, NY, USA) processed the data. Statistical analysis included the calculation of descriptive statistics consisting of mean values and standard deviations and frequencies among other measures. An analysis of differences between implant-supported grafts and sites occurred through ANOVA post hoc testing procedures. This study used a p-value of less than 0.05 as its statistical significance marker.

## Results:

A total of 85 patients were included in the study, comprising 42 males and 43 females, with a mean age of 48.6± 10.2 years. These patients received a total of 112 dental implants in previously grafted sites. The distribution of bone graft materials used showed that xenografts were the most frequently utilized (n=45; 40.2%), followed by allografts (n=39; 34.8%) and autografts (n=28; 25.0%). The overall implant survival rate at the one-year follow-up was 95.5%, with 107 out of 112 implants meeting the clinical success criteria-absence of mobility, pain, infection, or failure. The integration success rate of the grafts was 92.8% (104/112 sites). Table 1 (see PDF) summarizes the distribution and success rates of each graft type, while Table 2 (see PDF) presents the implant survival and graft success rates according to graft material. Autograft sites demonstrated the highest success rate among all groups, with 96.4% implant survival and graft success, followed by allografts (implant survival: 94.9%, graft success: 92.3%) and xenografts (implant survival: 95.5%, graft success: 91.1%). Marginal bone loss (MBL) at the 12-month follow-up was assessed radiographically. The autograft group showed the least amount of bone loss (0.96± 0.22 mm), followed by the allograft group (1.14± 0.25 mm), and the xenograft group (1.23± 0.31 mm). The difference in MBL among the three graft types was statistically significant (p < 0.05), as shown in Table 3 (see PDF). Further analysis based on implant location indicated that mandibular posterior sites exhibited the greatest marginal bone loss (1.23± 0.30 mm), while mandibular anterior sites had the lowest (1.01± 0.20 mm). However, the variation in bone loss among different implant locations was not statistically significant (p = 0.08). Survival rates across maxillary and mandibular regions remained consistently above 93%, as detailed in Table 4 (see PDF).

## Discussion:

This retrospective analysis demonstrates that bone grafting techniques succeed in protecting dental implants along with their surrounding bone structures. Implant therapy assisted by grafting techniques provides predictable outcomes based on previous research which showed success rates 90% to 98% in treated sites [1, 2]. Researchers observed a survival rate of 95.5%.The use of autogenous bone grafts demonstrated marginally better effectiveness toward integration success along with minimal marginal bone loss (MBL) based on published standards that recognize autografts as the benchmark treatment because of their ability to induce new bone growth [3, 4]. Research findings suggest that allografts and xenografts can achieve suitable clinical results although they are considered alternatives to the standard autogenous bone grafts when direct bone autograft procurement is not feasible [5, 6]. The health status of peri-implant tissue and the stability of implants in the long term depend on marginal bone loss measurements. The twelve-month MBL measurement came out to 1.12± 0.28 mm which supports implant success criteria stated by Aghaloo *et al.* that less than 1.5 mm of MBL indicates success during the first year [7]. Xenograft sites presented larger MBL values than receiving areas due to their delayed resorption speed and reduced bone remodeling capacity according to previous study findings [8, 9]. The placement position of dental implants affected marginal bone levels so posterior mandibular positions demonstrated higher amounts of bone resorption than anterior sites. Posterior region forces above the occlusion may impact long-term stability of the crestal bone according to research [10]. The healing process differs because of anatomical bone density and vascularity distinctions which exist between the maxilla and mandible [11]. Patients receive better implant-related outcomes when the surgeons incorporate proper surgical techniques and execute thorough post-operative care. The outcome of implant treatment strongly depends on flap design together with primary stability and proper timing of implant placement regarding grafting procedures [12]. Peri-implant tissue health together with stress distribution depends on the prosthetic protocol which includes abutment selection and restoration type [13]. The research findings show promise but the study contains specific limitations which need consideration. Research of this type faces limitations because its retrospective approach creates exposure to selection bias problems and incomplete documentation problems. The procedures to measure bone changes through x-rays possess protocol standards but they result in measurement errors from incorrect angulation positioning. The 12-month follow-up duration provides enough time for evaluating initial healing outcomes but cannot accurately demonstrate long-term modifications in bone stability according to research [14]. The existing study limitations do not change the proven effectiveness of bone grafting procedures in implant dentistry while showing both autologous and non-autologous grafts work effectively for treatment rehabilitation. More extensive research involving bigger participant size groups accompanied by extended observation periods should be conducted to authenticate these findings and better understand the biological characteristics of different graft products [15].

## Conclusion:

The success rate of dental implant therapy reaches high levels when patients undergo bone grafting surgery since this technique enables acceptable marginal bone loss along with excellent implant survival statistics regardless of graft material selection. The materials used for autogenous grafting achieved marginally higher success compared to other grafts although all options demonstrated clinical efficacy for maintaining extended implant stability.
